# A Facile Chemical Method Enabling Uniform Zn Deposition for Improved Aqueous Zn-Ion Batteries

**DOI:** 10.3390/nano11030764

**Published:** 2021-03-18

**Authors:** Congcong Liu, Qiongqiong Lu, Ahmad Omar, Daria Mikhailova

**Affiliations:** Leibniz Institute for Solid State and Materials Research (IFW) Dresden e.V., 01069 Dresden, Germany; congcong.liu@ifw-dresden.de (C.L.); a.omar@ifw-dresden.de (A.O.)

**Keywords:** zinc metal anode, copper coating, alloy interfacial layer, uniform Zn deposition, aqueous zinc-ion battery

## Abstract

Rechargeable aqueous Zn-ion batteries (ZIBs) have gained great attention due to their high safety and the natural abundance of Zn. Unfortunately, the Zn metal anode suffers from dendrite growth due to nonuniform deposition during the plating/stripping process, leading to a sudden failure of the batteries. Herein, Cu coated Zn (Cu–Zn) was prepared by a facile pretreatment method using CuSO_4_ aqueous solution. The Cu coating transformed into an alloy interfacial layer with a high affinity for Zn, which acted as a nucleation site to guide the uniform Zn nucleation and plating. As a result, Cu–Zn demonstrated a cycling life of up to 1600 h in the symmetric cells and endowed a stable cycling performance with a capacity of 207 mAh g^−1^ even after 1000 cycles in the full cells coupled with a V_2_O_5_-based cathode. This work provides a simple and effective strategy to enable uniform Zn deposition for improved ZIBs.

## 1. Introduction

The significantly growing consumption of fossil fuels worldwide has led to severe global warming. To reduce the usage of fossil fuels and sustainably develop renewable energy utilization, e.g., solar energy and wind energy, advanced energy storage systems (such as batteries and supercapacitors) are in high demand [1,2,3,4]. For example, lithium-ion batteries (LIBs) have seen tremendous success as one of the most common types of power source in the portable electronics market, due to their high energy density [5,6,7]. However, the fire risk of the flammable organic electrolyte, high cost, as well as limited reserves of lithium, severely restrict the largescale implementation of LIBs in automotive and stationary storage applications [8,9]. In this regard, developing alternative battery technology is important and essential. Recently, rechargeable aqueous Zn-ion batteries (ZIBs) have been increasingly investigated, due to their low cost, the high theoretical capacity of zinc (819 mAh g^−1^), and the low redox potential (−0.762 V vs. SHE). More importantly, the aqueous electrolyte possesses the merits of nonflammable, nontoxic, and environmental benignity [10,11,12]. However, ZIBs suffer from a challenging issue related to the zinc anode, which is afflicted with uncontrollable dendrite formation during the Zn stripping/plating process. The problem is generally attributed to what is known as the “tip effect”, where, due to charge aggregation near the protuberances on the inhomogeneous zinc surface, zinc ions are easily absorbed onto the protruded tips and nucleate preferentially on these spots, thereby triggering continuous growth and finally form Zn dendrites [13,14,15,16].

To address this issue, various methods have been developed, such as optimizing the electrolytes [17], stabilizing the structure by employing host materials [18], and modifying the surface [19,20,21,22,23,24]. Among surface modification methods, an efficient strategy to stabilize the Zn anode during the stripping/plating process is a surface coating with a metal such as In [19], Au [20], Ag [21], and Cu [21] coating, which acts as a nucleation site to guide uniform Zn deposition. For instance, Zhang et al. developed Ag and Cu coatings on Zn metal through the thermal evaporation method [21]. They found out that Ag−Zn and Cu–Zn alloys were formed after cycling, which improved the affinity to Zn and further contributed to the uniform nucleation and deposition of Zn. Owing to its easy alloy formation with Zn and low cost, Cu is a promising material for Zn stabilization as compared with noble metals such as Ag and Au. However, the high temperature required in the thermal evaporation method would not enable cost efficiency and would limit largescale applicability. Consequently, developing direct, economically viable methods to fabricate an effective Cu coating on Zn metal is required for the practical application of ZIBs. From the point of view of efficacy and cost, chemical methods may be a good alternative. Therefore, Cu coating on Zn metal prepared by a chemical route to guide uniform Zn nucleation and deposition is a meaningful route to explore.

In this work, Cu coated Zn (Cu–Zn) was prepared by a facial chemical method. Cu coating was transformed to Cu–Zn alloy after cycling, which acted as the nucleation sites to guide uniform Zn nucleation and deposition. As a result, Cu–Zn not only showed improved cycling life in the symmetric cells, but also enabled a stable cycling performance in the full cells coupled with V_2_O_5_-based cathode. The results demonstrated that our strategy was facile and efficient to guide uniform Zn deposition for improved ZIBs.

## 2. Materials and Methods

### 2.1. Preparation of the Cu Coated Zn

A pristine zinc foil (125 μm in thickness, Goodfellow GmbH, Bad Nauheim, Germany) was polished with sandpaper and then cut into discs with a diameter of 12 mm. A portion of 100 µL of 0.1 M copper sulfate (CuSO_4_, 99.99%, Sigma-Aldrich, St Louis, MO, USA) aqueous solution was dropped on the zinc disc surface and kept for 3 min to get a one-side-coated Zn foil. The treated zinc metal was washed with deionized water a few times and stored in the air to dry naturally.

### 2.2. Preparation of Poly(3,4-ethylenediophene)-Coated V_2_O_5_ (V_2_O_5_-PEDOT) Cathode

For the synthesis of V_2_O_5_-PEDOT, 7 g of commercial V_2_O_5_ powder (Sigma-Aldrich, St Louis, MO, USA) was dispersed in 70 mL of deionized water, then 1 mL of 3,4-ethylenediophene (EDOT, Aladdin, Shanghai, China) was added dropwise. The mixture was continuously stirred for 6 days and filtered. The obtained powder was dried in a vacuum oven at 70 ℃ for overnight [25]. The cathode was prepared by coating the slurry of V_2_O_5_-PEDOT, Super C65 (TIMCAL, Bodio, Switzerland), and polyvinylidene fluoride (PVDF, Solef 21216, Solvay, Milan, Italy) in N-methyl pyrrolidone (99%, NMP, Sigma-Aldrich, St Louis, MO, USA) at a mass ratio of 8:1:1 on to stainless steel mesh (500 pores per linear inch, wire diameter of 0.2 mm, Gelon lib group, Linyi, China) and dried in a vacuum at 80 °C overnight. The mass loading of V_2_O_5_-PEDOT was about 1 mg∙cm^−1^.

### 2.3. Characterization

A scanning electron microscope (FFG-SEM, Zeiss-Leo Gemini 1530, Carl Zeiss NTS GmbH, Oberkochen, Germany) was employed to characterize the surface morphologies of zinc electrodes at different stages of the experiment process. Elemental mappings were performed using energy dispersive X-ray spectroscopy with a Bruker XFlash 6 detector (Bruker, Karlsruhe, Germany). X-ray diffraction (XRD) was carried out on a Panalytical X’pert Pro diffractometer device (Panalytical, Almelo, Netherlands) operating with Co Kα radiation in reflection mode. The X-ray photoelectron spectroscopy (XPS) analysis was performed in a PHI 1600 ESCA (PerkinElmer, Waltham, MA, USA) spectrometer with a monochromatic Al-Kα source. The binding energies were calibrated using the C 1s peak at 284.8 eV.

### 2.4. Electrochemical Characterization

The ZIBs were assembled using Swagelok cells under ambient conditions in air. 3 M Zn (CF_3_SO_3_)_2_ (98%, Sigma-Aldrich, St Louis, MO, USA) aqueous solution and glass fiber (Whatman GF/D, Whatman, Clifton, NJ, USA) were used as electrolyte and separator, respectively. The galvanostatic cycling of symmetric cells was performed with different current densities using LAND CT2001A (Wuhan Land Electronic Co., Ltd., Wuhan, China) potentiostat. The galvanostatic cycling performance of full cells was tested in the voltage range of 0.3 V ≤ U ≤ 1.6 V vs. Zn/Zn^+^ using LAND potentiostat.

## 3. Results

**Figure 1:**
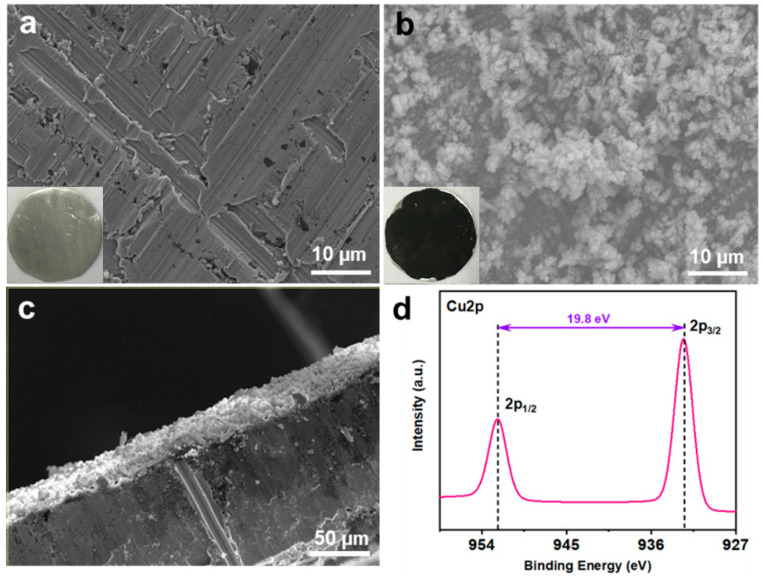
SEM images and optical images (insets) of (**a**) bare Zn foil and (**b**) Cu–Zn foil, (**c**) cross-section SEM images of Cu–Zn foil, (**d**) high resolution Cu 2p XP spectrum measured on Cu–Zn foil.

**Figure 2:**
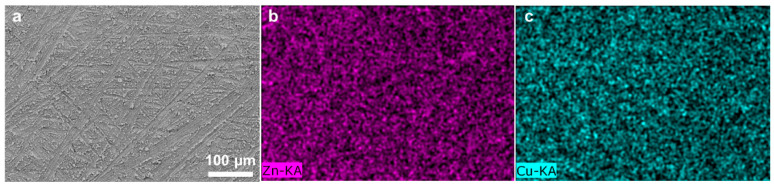
(**a**) SEM image and (**b**,**c**) the corresponding elemental mappings of Cu–Zn.

Cu-coated Zn (Cu–Zn) was prepared via an in situ chemical method by dropping an aqueous CuSO_4_ solution on bare zinc foils. The treated Zn foils were subsequently washed and dried. Due to the potential difference between Zn^2+^/Zn and Cu^2+^/Cu, a spontaneous replacement reaction occurred and the surface of Zn changed to a black color after treatment (insets of Figure 1a,b). Scanning electron microscopy (SEM) was conducted to characterize the morphology of bare Zn and Cu–Zn. The bare zinc had a rough and scratched surface with a unique texture due to the polish process (Figure 1a). Cu–Zn clearly showed a coated surface (Figure 1b). The elemental mapping of Cu–Zn showed Cu uniformly distributed on the surface of Zn (Figure 2). The thickness of the Cu coating layer was about 20 µm by the cross-section SEM image (Figure 1c). The high resolution Cu 2p XP spectrum of Cu–Zn showed two peaks located at 932.5 eV and 952.3 eV, assigned to metallic Cu (Figure 1d) [26]. It should be noted that it was challenging to distinguish between the metallic Cu and Cu(I) by binding energy of Cu 2p, as the Cu 2p signals overlapped. Although the Cu LMM Auger peak is recommended to be used for the identification and analysis of Cu(I), unfortunately, the intensity of the signal in the Cu LMM Auger region of Cu–Zn was too weak for further analysis [27]. However, in our case since the reaction represented a chemical reduction of Cu(II), the formation of Cu(I) was highly unlikely.

**Figure 3:**
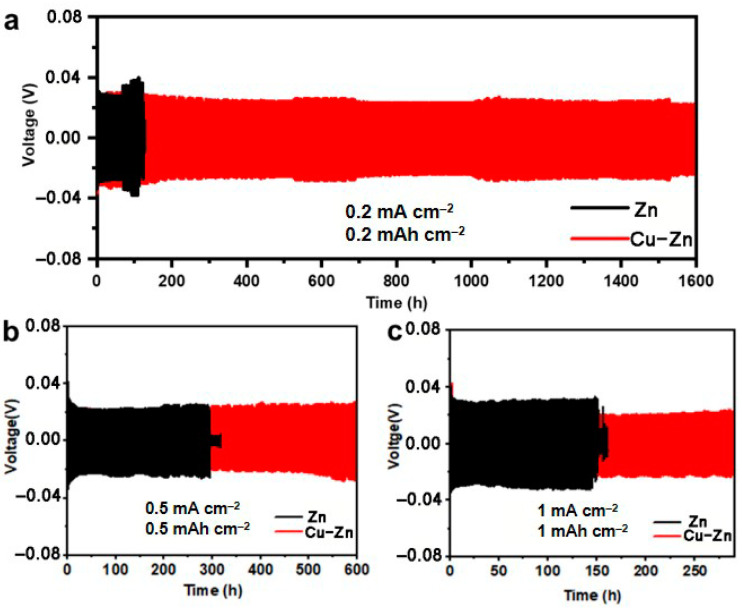
Long-term galvanostatic discharge/charge profiles of symmetric cells with bare Zn and Cu–Zn at current density of (**a**) 0.2 mA cm^−2^, (**b**) 0.5 mA cm^−2^, and (**c**) 1 mA cm^−2^.

In order to evaluate the effect of the Cu coating on the Zn plating/stripping behavior, symmetric cells comprised of bare Zn and Cu–Zn (henceforth referred to Zn//Zn and Cu-Zn//Cu–Zn respectively) were assembled and tested. Galvanostatic charge/discharge was performed at different current densities. At 0.2 mA cm^−2^, the Zn//Zn cell exhibited an increasing overpotential after 70 h cycling, subsequently showing an abnormal voltage drop at 110 h, indicating a short circuit of the battery due to the zinc dendrite. In contrast, at 0.2 mA cm^−2^, Cu-Zn//Cu–Zn showed a markedly prolonged cycling life of over 1600 h with a lower overpotential (Figure 3a). Even when the current density was increased to 0.5 mA cm^−2^ and 1 mA cm^−2^, Cu-Zn//Cu–Zn cells showed a prolonged cycle life of at least 600 h and 290 h, respectively, whereas Zn//Zn cells exhibited a cycling life of only 300 h and 145 h, respectively (Figure 3b,c). Moreover, the cycling life of Cu–Zn symmetric cells was much better than that for most of the reported works (Table 1). The results highlight the effectiveness of the Cu-coating strategy via the chemical route towards improving the cycling stability and prolonging the cycle life, while at the same time involving a rather facile process.

**Figure 4:**
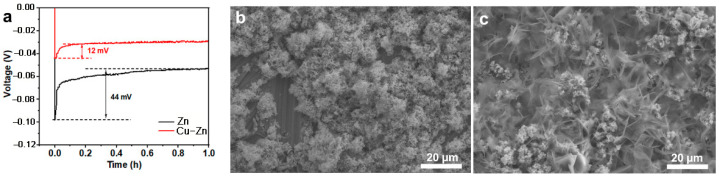
(**a**) The voltage-time curves during Zn nucleation and deposition on Zn and Cu–Zn at 1 mA cm^−2^, SEM images of (**b**) Zn and (**c**) Cu–Zn after Zn-depositing with a capacity of 2 mAh cm^−2^ at current density of 1 mA cm^−2^.

The nucleation overpotential is related to the kinetics of the Zn nucleation and deposition process, and the Zn nucleation consequently determines the quality of Zn deposition [28]. Thus, the Zn nucleation overpotential was measured and compared. As shown in Figure 4a, Cu–Zn showed a lower nucleation potential of 12 mV as compared to 44 mV for Zn, indicating a lower nucleation barrier for Cu–Zn which contributed to a uniform Zn nucleation. To further study the effect of Cu coating on the Zn deposition, the morphology of Cu and Cu–Zn after Zn deposition was characterized (Figure 4b,c). Bare Zn shows a huge amount of Zn microclusters with a porous structure, demonstrating uneven deposition. In contrast, Cu–Zn exhibited a dense and uniform Zn deposit, confirming the beneficial role of the Cu coating.

**Figure 5:**
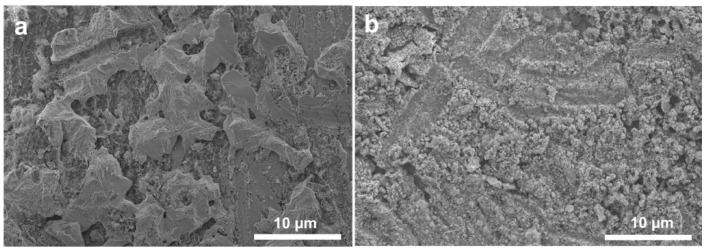
SEM images of (**a**) Zn and (**b**) Cu–Zn after 30 cycles at a current density of 5 mA cm^−2^ with a capacity of 1 mAh cm^−2^.

**Figure 6:**
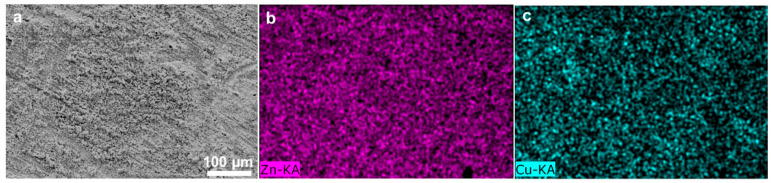
(**a**) SEM image and (**b**,**c**) corresponding elemental mappings of Cu–Zn after 50 cycles at current density of 5 mA cm^−2^ with a capacity of 1 mAh cm^−2^.

**Figure 7:**
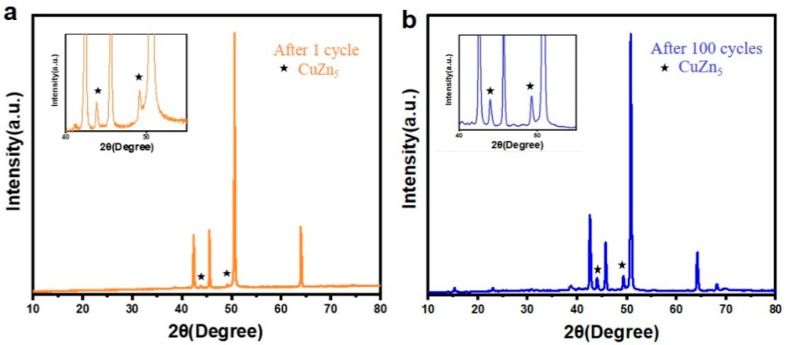
XRD pattern of Cu–Zn (**a**) after 1 cycle and (**b**) after 100 cycles at current density of 5 mA cm^−2^ with a capacity of 1 mAh cm^−2^. ★ represents the XRD peak of CuZn_5_.

To further investigate the Zn electrodeposition behavior, the morphology of Zn and Cu–Zn electrodes after 30 cycles at a high current density of 5 mA cm^−2^ was characterized (Figure 5a,b). Bare Zn showed a bulk Zn deposition morphology while Cu–Zn exhibited a uniform and compact morphology. Elemental mapping of Cu–Zn after 50 cycles at a high current density of 5 mA cm^−2^, confirmed that the uniform distribution of the Cu coating was maintained (Figure 6). It was expected that Cu–Zn alloy was formed during the stripping/plating, due to the negative Gibbs free energy of the reaction [29,30]. In order to check this, a Cu–Zn electrode after 1 cycle was characterized by XRD. As is shown in Figure 7a, two weak peaks at 2θ = 44.1° and 49.5° additionally appeared in the XRD pattern of Cu–Zn after cycling, corresponding to the CuZn_5_ phase (PDF Number 00-035-1152). The XRD pattern of Cu–Zn after 100 cycles showed that the Cu–Zn alloy was retained, confirming the durability of Cu–Zn alloy during cycling (Figure 7b). The binding energy of Zn-CuZn_5_ (−1.94 eV) was higher than that of Zn-Cu (−1.58 eV), demonstrating a high Zn affinity of the formed CuZn_5_ [30]. Thus, the formed alloy could effectively reduce the activation energy of zinc nucleation and the plating resistance of zinc. Consequently, the zinc grew in a smaller size and achieved uniform nucleation without the formation of long and disordered dendrites.

**Figure 8:**
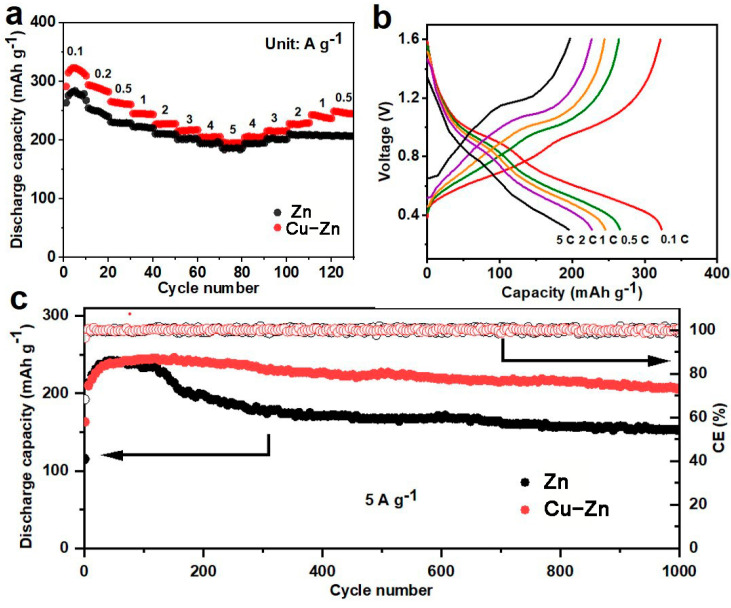
(**a**) Rate performance of V_2_O_5_-PEDOT//Zn and V_2_O_5_-PEDOT//Cu-Zn, (**b**) charge/discharge curves of V_2_O_5_-PEDOT//Cu–Zn at different current densities, (**c**) cycling performance of V_2_O_5_-PEDOT//Zn and V_2_O_5_-PEDOT//Cu–Zn at a current density of 5 A∙g^−1^.

In order to validate the practical application of Cu–Zn, it is necessary to evaluate full cells with cathodes. V_2_O_5_-based materials have been used as the cathode for ZIBs due to their high capacity, and V_2_O_5_-PEDOT offers improved and stable performance [25,31,32,33,34]. Therefore, V_2_O_5_-PEDOT was synthesized based on previously reported work, and full batteries where Zn anodes coupled with V_2_O_5_-PEDOT cathodes were assembled and tested (henceforth referred to as V_2_O_5_-PEDOT//Zn and V_2_O_5_-PEDOT//Cu–Zn, accordingly). The rate performance of the full cells was investigated, as shown in Figure 8a,b. At 0.1, 0.2, 0.5, 1, 2, 3, 4, 5 A∙g^−1^, V_2_O_5_-PEDOT//Cu–Zn cell revealed a high capacity of 323, 311, 266, 246, 227, 215, 205, 195 mAh g^−1^ respectively, while the V_2_O_5_-PEDOT//Zn battery showed a capacity of 284, 255, 231, 223, 211, 201, 194, 187 mAh g^−1^, respectively. When the current density was set back to 0.5 A∙g^−1^, the capacity of the V_2_O_5_-PEDOT//Zn battery only reached a value of 208 mAh g^−1^. In contrast, the capacity of the V_2_O_5_-PEDOT//Cu–Zn battery recovered significantly to a capacity of 250 mAh g^−1^, indicating high reversibility. The long-term cycling was also tested at the current density of 5 A∙g^−1^ (Figure 8c). During the first 60 cycles, both cells showed an enhancement of capacity due to activation. V_2_O_5_-PEDOT//Zn underwent a capacity fade after 120 cycles, which was ascribed to the formation of zinc dendrites resulting in “dead Zn” with a subsequent cycling, thereby increasing the internal resistance of the batteries. In sharp contrast, V_2_O_5_-PEDOT//Cu–Zn showed a relatively high and stable capacity over 1000 cycles. The stable cycling performance of the full cells with Cu–Zn anodes highlighted the efficiency of Cu in suppressing zinc dendrite and “dead Zn” formation by guiding uniform Zn deposition, and demonstrated high potential for practical applications.

## 4. Conclusions

In conclusion, we developed a Cu-coated Zn by a straightforward CuSO_4_ aqueous solution treatment strategy. A CuZn_5_ alloy was formed after cycling in the battery, which guided the uniform Zn nucleation suppressing the formation of large size Zn dendrites thus improving cycling stability. This strategy not only enabled a remarkable improvement in the cycling life of symmetric cells, but also endowed a high capacity and a stable cycling performance of the full cells coupled with the V_2_O_5_-PEDOT cathode. Therefore, coupled with the easy and scalable Zn treatment route, the approach is highly viable for practical implementation in ZIBs. Moreover, this work should open up a promising direction for modifying and protecting other metallic electrodes of rechargeable aqueous battery systems.

## Figures and Tables

**Table 1:** Comparison of the performance of Cu–Zn symmetric cells with recent literature on various Zn surface modification strategies.

Protective Layers	Current Density (mA cm^−2^)	Capacity (mAh cm^−2^)	Life (h)	Reference
	0.2	0.2	1600	
Cu coating	0.5	0.5	600	This work
	1	1	290	
In coating	0.2	0.2	1500	[19]
Au coating	0.25	0.05	2000	[17]
MXene	0.2	0.2	800	[22]
CaCO_3_ coating	0.25	0.05	840	[24]
TiO_2_ coating	1	1	150	[23]

## Data Availability

Data is contained within the article.
